# Radioprotective potential of melatonin against ^60^Co γ-ray-induced testicular injury in male C57BL/6 mice

**DOI:** 10.1186/s12929-015-0156-9

**Published:** 2015-07-24

**Authors:** Shahanshah Khan, Jawahar Singh Adhikari, Moshahid Alam Rizvi, Nabo Kumar Chaudhury

**Affiliations:** Chemical Radioprotector and Radiation Dosimetry Research Group, Division of Radiation Biosciences, Institute of Nuclear Medicine and Allied Sciences, Defence Research & Development Organization, Brig. S. K. Mazumdar Road, New Delhi, Delhi 110054 India; Genome Biology Laboratory, Department of Biosciences, Faculty of Natural Sciences, Jamia Millia Islamia, New Delhi, 110025 India

**Keywords:** Melatonin, γ-irradiation, DNA strands breaks, ATM, TAC, Spermatogenic cells, Sperm abnormalities

## Abstract

**Background:**

Melatonin, the chief secretary product of pineal gland, is a strong free radical scavenger and antioxidant molecule. The radioprotective efficacy and underlying mechanisms refer to its antioxidant role in somatic cells. The purpose of this study, therefore, was to investigate the prophylactic implications of melatonin against γ-ray-induced injury in germinal cells (testes). C57BL/6 male mice were administered melatonin (100 mg/kg) intra-peritoneally 30 min prior to a single dose of whole-body γ-irradiation (5 Gy, 1 Gy/minute) using ^60^Co teletherapy unit. Animals were sacrificed at 2h, 4h and 8h post-irradiation and their testes along with its spermatozoa taken out and used for total antioxidant capacity (TAC), lipid peroxidation, comet assay, western blotting and sperm motility and viability. In another set of experiment, animals were similarly treated were sacrificed on 1^st^, 3^rd^, 7^th^, 15^th^ and 30^th^ day post-irradiation and evaluated for sperm abnormalities and histopathological analysis.

**Results:**

Whole-body γ-radiation exposure (5 Gy) drastically depleted the populations of spermatogenic cells in seminiferous tubules on day three, which were significantly protected by melatonin. In addition, radiation-induced sperm abnormalities, motility and viability in cauda-epididymes were significantly reduced by melatonin. Melatonin pre-treatment significantly inhibited radiation-induced DNA strands breaks and lipid peroxidation. At this time, radiation-induces activation of ATM-dependent p53 apoptotic proteins-ATM, p53, p21, Bax, cytochrome C, active caspase-3 and caspases-9 expression, which were significantly reversed in melatonin pre-treated mice. This reduced apoptotic proteins by melatonin pre-treatment was associated with the increase of anti-apoptotic-Bcl-x and DNA repair-PCNA proteins in irradiated mice. Further, radiation-induced decline in the TAC was significantly reversed in melatonin pre-treated mice.

**Conclusions:**

The present results indicated that melatonin as prophylactic agent protected male reproductive system against radiation-induced injury in mice. The detailed study will benefit in understanding the role of melatonin in modulation of radiation-induced ATM-dependent p53-mediated pro-vs.-anti apoptotic proteins in testicular injury. These results can be further exploited for use of melatonin for protection of male reproductive system in radiotherapy applications involving hemibody abdominal exposures.

**Electronic supplementary material:**

The online version of this article (doi:10.1186/s12929-015-0156-9) contains supplementary material, which is available to authorized users.

## Background

Whole-body radiation exposure can cause reversible or permanent damages in male reproductive system [1]. Testis is one of most radiosensitive reproductive organs, sensitive to radiation dose as low as 0.1 Gy, because of highly proliferating spermatogonial cells [2, 3]. Significant decrease in sperm count and morphological abnormalities has been reported at radiation doses as low as 1-2 Gy in rats. Testes possesses germ cells at different stages of development, a process known as spermatogenesis, and developing sperms are very sensitive to ionizing radiation, known to affect morphology, function and ultimately the spermatogenesis [4, 5]. Spermatogenesis is affected after radiation as both Leydig and sertoli cells die during cell division. Radiation doses required to kill spermatocyte is higher than spermatogonia, eventually lead to disappearance of spermatids, spermatogonia and spermatocyte. In human, lowering of sperm count and temporary azoospermia was reported at radiation doses as low as 0.3 Gy [6, 7]. Proliferation of Leydig and sertoli cells is inhibited after exposure to radiation dose of 1 Gy. Recovery and re-population will depend on proliferation of the surviving stem cell that is spermatogonia and ultimately the germinal epithelium with germ cells [6, 7]. Whole body radiation exposure in case of accidental exposure, overexposure among radiation workers and abdominal irradiation in radiotherapy for example in Hodgkin disease, may receive radiation doses harmful for testes. Therefore, it is desired that the promising radioprotector to have efficacy for protection of reproductive systems.

A number of approaches for development of radioprotectors are under investigations in different laboratories. Radiation exposure from low LET γ-radiation, gamma radiation generates free radicals in cells by radiolysis of waters in cells. These free radicals are reactive oxygen species, highly damaging for all biomolecules, DNA, proteins and lipids in cells. The biological manifestations are in the form of radiation-induced injuries in various organs with increasing radiation doses [8–10]. Antioxidants are known to scavenge free radicals, and therefore considered strong candidates for development of radioprotector [11]. Several studies have emphasised on antioxidant properties of pure compounds and complex mixture of molecules in extracts from plants. Most of these studies have focussed on search for new molecules validated through enhanced survival and supported by the role of antioxidant properties and related mechanisms. Protection of radiosensitive organs including testis is rational for developing radioprotectors for planned whole body or hemi body exposures. A number of investigations in recent past have recommended melatonin for development of radioprotector [12]. Vijayalaxmi et al. performed a series of studies through *in-vivo*, *in-vitro* using somatic cells and *ex-vivo* using human blood and demonstrated potential of melatonin for development of radioprotector [12–22]. These studies were primarily focussed on ability of melatonin on reducing radiation-induced DNA damage using cytogenetic assays for chromosomal aberrations, micronuclei in human peripheral blood, bone marrow of mice and the results strongly demonstrated the ability of melatonin pre-administration on protection of genetic damages. Melatonin has not been investigated in detailed for its radioprotective efficacy in lowering radiation-induced injuries and recovery in testis. Few studies have reported melatonin mediated protection of germinal cells in testes of whole-body irradiated rodents. These studies have depicted the radioprotective effects of melatonin pre-treatments by morphological and ultra-structural studies at single time point following whole-body/partial radiation exposure [23–25]. In addition, few studies have reported radioprotective effects of herbal extracts from different plants on testes in rodents [26–28].

Melatonin is an endogenous chief secretary product of pineal gland, has strong antioxidant and free radical scavenging properties [29]. Antioxidant properties of melatonin are well documented and understood at both molecular and cellular levels [30–32]. Melatonin up-regulates several anti-oxidatant enzymes (catalase, GSH, SOD) and down-regulates pro-oxidant enzyme (nitric oxide synthase), and thus protects cellular biomolecules from radiation-induced oxidative damages [12, 33, 34]. Further, melatonin facilitates repair processes of radiation-induced DNA damages via its stimulatory action on different repair enzymes [12, 35]. Whole body or partial body exposure has potential threat for germ cells population, but literature on radioprotection by melatonin in reproductive organ is scanty and, therefore, required more attention for investigation its role in reproductive system.

Since absorbed radiation dose in accidental sites and in planned exposure scenarios is less likely to be lethal and, therefore relevant investigation on the effect of sub-lethal radiation doses require more attention. In the present study, therefore, we have investigated the radioprotective potential of melatonin, for its ability to rescue germinal cells (testes) injuries induced by γ-irradiation at sub-lethal dose of 5 Gy in C57BL/6 male mice. The objective was to study the detailed histological and morphological abnormalities along with the mechanism of radioprotection by melatonin in germinal cells with emphasis on ATM-mediated pathways in C57Bl/6 male mice, a recommended animal model for development of radioprotector [36]. We have evaluated the radiation-induced qualitative and quantitative histopathological changes, total antioxidant capacity (TAC), lipid peroxidation, DNA strands breaks and ATM-dependent pro-versus.-anti apoptotic proteins expression in testes. In addition, radiation-induced morphological sperm abnormality, motility and viability were also measured. The results of this study have demonstrated that melatonin provided radioprotection in testes of mice when pre administered intra-peritoneally as a single dose 30 min prior to the whole-body irradiation. Melatonin pre-treatment has increased TAC and decreased lipid peroxidation, DNA strands breaks as well as sperm abnormality leading to recovery of spermatogenic cell population in irradiated testes. The mechanism of protection by melatonin involve inhibition of radiation-induced expression of ATM, p53, p21, Bax, Bcl-x, cytochrome C, active caspases-3 and caspases-9. The results can be useful for further validation studies in higher models for development of radioprotector for planned exposure.

## Methods

### Antioxidants and chemicals

Melatonin (N-acetyl-5-methoxytryptamine), soybean oil, Bradford reagent, PMSF, BSA, propidium iodide, protease inhibitor cocktail, anti-p53, anti-Bax, anti-Bcl-x, HRP-conjugate and formalin were procured from Sigma-Aldrich Chemical Co., St. Louis, MO, USA. EGTA, tris-HCL, trichloroacetic acid, tween-20, tween-100, skimmed milk powder, and phosphate buffer saline (PBS) were purchased from HiMedia, Mumbai, India. EDTA, NaCl ABTS (2,2′-azinobis (3-ethylbenzothiazoline-6-sulfonate), and ethanol were from Merck, Germany. ECL chemiluminescence reagent was from Amersham Pharmacia Biotech, Piscataway, NJ, USA. Dimethyl sulfoxide (DMSO) and sodium dodecyle sulphate were from Calbiochem, San Diego CA USA. DNase free RNase was procured from Genei, Bangalore, India.

### Animal model

Male C57BL/6 (8-9 week-old) mice were issued from animal facility one week prior to acclimatization. Six mice were housed in polypropylene cage with sterile paddy husk as bedding and certified sterile food as well as acidified water *ad libitum* throughout the experiment. All cages were placed in a pre-maintained room (light/dark cycle 12-h, temperature 23 ± 2 °C and relative humidity 55 ± 5 %). Mice received no treatment (drug or radiation) served as sham control. The group of mice received 100 mg/kg body weight of melatonin served as melatonin control. Radiation alone treated mice received 5 Gy (1 Gy/minute) whole-body γ-irradiation, whereas melatonin pre-treated mice received 100 mg/kg body weight intra-peritoneally 30 min prior to 5 Gy (1 Gy/minute) whole-body γ-irradiation. The protocols used in this experiment were approved by the Institutional Animal Ethics Committee (Institutional Animal Ethics committee approval number is INM/IEAC/2012/06). All experimental procedures were practised to minimize suffering during sacrifice of animal through cervical dislocation.

### Melatonin preparation and administration

Melatonin was freshly prepared in soybean oil. A prophylactic single dose of melatonin (100 mg/kg body weight) in a volume of 0.2 ml was administered intra-peritoneally using sterile 26-gauge needle 30 min prior to whole-body γ-irradiation.

### Gamma-irradiation

Animals were exposed to 5 Gy whole body γ-irradiation (dose rate 1 Gy/minute) using a ^60^Co Teletherapy unit (Bhabhatron-II, Panacea, India). The dose rate was calibrated by physical dosimetry by radiation safety team of the Institute as a part of routine calibration requirement in accordance to the Atomic Energy Regulatory Agency, India.

### Histological examination in testes

To determine the effect of whole-body γ**-**irradiation in testes, right testes of individual mice was dissected out on 1^st^, 3^rd^, 7^th^, 15^th^ and 30^th^ days after irradiation, and extra tissues were removed in pre-chilled PBS and fixed in 10 % formalin at room temperature. After embedding 5μM thick sections were cut, stained with hematoxylene and eosin (H & E) and mounted. To determine the effect of γ**-**irradiation on quantitative changes in testes, five sections were scored per slide with ten seminiferous tubules in each section for a total of 5 × 10 seminiferous tubules per mouse. Fifteen mice were randomly divided in five groups and three mice were considered for each five time points to generate mean number of spermatogonia, sertoli cell, spermatocyte and spermatids. All slides were coded by individual person not involved in scoring and after completion of all slides codes were opened.

### Morphological examination of mouse sperm

Animals were scarified on 1^st^, 3^rd^, 7^th^, 15^th^ and 30^th^ days post-irradiation. Both caudal epididymes were dissected out, cleaned in pre-chilled PBS and minced with fine curved scissor into 2 ml of TNE buffer (0.15 M NaCl, 0.01 M Tris-HCL, 0.001 M EDTA, pH 7.4) on ice. Aliquot was filtered through 100 μm nylon mess strainer (BD Biosciences, San Diego, CA, USA) to remove tissue fragments. A drop of aliquot was transferred to heamocytometer (Neubauer, Marienfeld, Germany) and observed under inverted microscope (4200, Meiji, Japan) to insure the integrity and density of cells (1 × 10^6^ cells/ml).

To evaluate the γ**-**irradiation-induced sperm abnormalities, slides were prepared on 1^st^, 3^th^, 7^th^, and 15^th^ days post-irradiation. A small volume of cell suspensions (1 × 10^6^ cells/ml) were transferred with Pasteur pipette onto pre-marked cleaned glass slide and a thin smear was made by using edge of another glass slide. The smear was air dried and fixed by dipping slide into ethanol (80 % v/v) for few seconds. The slide was again air dried and stained with 1 % Eosin-Y for 30 min at room temperature. After staining, slides were washed with Milli-Q water for few seconds and air dried. For each mouse, at least 1000 sperms were examined using upright motorized compound microscope with DIC attached and digital imaging system (Axio Imager M2, Zeiss, Germany) at 400X magnification for normal and abnormal (hook less, banana-like, amorphous, folded, short tail, two tail and two head) forms. Three mice were used per group and a total of 3 × 1000 sperms were expressed in percentage. All slides were coded for avoiding scorer biasness.

### Sperm motility test

To assess the effect of irradiation on motility of sperm cells after 2h, 4h and 8h post-irradiation, 10-15 μL single cell suspensions (1 × 10^6^ cells/ml) were transferred to haemocytometer and observed under inverted microscope. All sperms were observed individually under microscope with original magnification of 400X and considered as motile if they had shown any movement. Each sample was counted at least three times. Results presented in percentage sperm motility index was determined by dividing total number of motile sperm with sum of motile plus non-motile sperm.

### Sperm viability test

To determine the affect of whole-body γ**-**irradiation on viability after 2h, 4h and 8h post-irradiation, 90 μl of sperm suspensions (1 × 10^6^ cells/ml) were mixed with 10 μl of 1 % Eosin-Y and after 3 to 4 min both stained and unstained cells were counted using heamocytometer with (400X original magnification) inverted microscope. Each sample was examined at least three times for unstained as well as stained cells and presented in proportion (%) of eosin negative (unstained or viable) sperms.

### Total antioxidant capacity in testes

Animals were killed by cervical dislocation at 2h, 4h, and 8h post-irradiation. Testes were cleaned in pre-chilled PBS on ice and weighed using balance (CPA225D, Sartorius, Göttingen). Testes homogenates were prepared in pre-chilled PBS (10 % w/v) by tissue homogenizer (OMNI, TH, USA) and centrifuged with 12000 g at 4 °C for 15 min. Supernatants were stored immediately at -80 °C for further analysis, if not used on same day.

Total antioxidants capacity (TAC) of testes was determined spectrophotometricaly by ABTS radical scavenging assay [37]. In brief, this assay involves the production of blue/green ABTS^•**+**^chromophore (ABTS^•**+**^ radical cation) by mixing ABTS (7 mM) with potassium persulfate (2.45 mM) in water and kept in dark at room temperature for 12 to 16 h. The ABTS^•**+**^ chromophore has absorption maxima at 645 nm, 734 nm and 815 nm wavelengths [38]. The working solution of ABTS^•**+**^ was prepared by diluting the ABTS^•**+**^ stock solution in PBS to the absorbance of 0.70 ± 0.02 at 734 nm. For ABTS^•**+**^ assay, 20 μL of biological sample was mixed with 2 ml of working ABTS^•**+**^ solution in disposable plastic cuvette. The decrease in ABTS^•**+**^ radical absorbance was monitored till 30 min in kinetic mode at 734 nm using UV-visible spectrophotometer (Cary100Bio, Varian, Australia.). The percentage inhibition of individual sample was calculated and equated with Trolox standard curve obtained under the similar experimental situation (1-32 μM final concentration).

Protein estimation of these samples was performed by Lowry method using BSA standard curve following the manufacturer’s instruction (Protein estimation kit, GeNei^TM^, Merck). The Trolox equivalent ABTS^•**+**^ radical scavenging capacities of testes was expressed as μM Trolox equivalent (TE) /μg protein. The amount of Trolox (μmol) is an equivalent to 1 μg of protein.

### Thiobarbituric acid reactive substances assay in testes

Thiobarbituric acid reactive substances **(**TBARS) productions in testicular germ cells were measured at 2 h, 4h, and 8h post-irradiation. Testes were homogenized in pre-chilled PBS (10 % w/v) using tissue homogenizer (OMNI TH, USA). TBARS level in testicular germ cells were measured following the standard protocol described elsewhere [39]. TBARS were represented as nmol per mg of protein.

### Alkaline comet assay in testes

Testes were minced with fine curved scissor in pre-chilled PBS on ice at 2h, 4h and 8h post-irradiation. Single cells suspensions were obtained by filtering the aliquot with 100 μM nylon mesh strainer (BD Biosciences, San Diego, CA, USA). The number of germ cells (1 × 10^6^ cells/mL) was maintained by heamocytometer (Neubauer, Marienfeld, Germany) using inverted microscope (4200, Meiji, Japan).

Alkaline comet assay was performed to assess the DNA strand breaks in germ cells following the guidelines developed by Tice and co workers [40]. Briefly, single cells suspensions were mixed with 0.7 % (w/v) low-melting-point agarose and immediately pipette onto a pre-coated comet slide with 1 % (w/v) normal-melting-point agarose. The slides were transferred onto slide tray resting on ice packs for harden agarose layer (at least 10 min) and then immersed in a pre-chilled lysing solution (2.5 M NaCl, 100 mM Na_2_EDTA, 10 mM Tris, 1 % SLS, pH 10) containing 10 % (v/v) DMSO and 1 % (v/v) Triton X 100 overnight at 4 °C. After complete lysis, gently remove slide and placed in pre-chilled unwinding solution (200 mM NaOH, 100 mM Na_2_EDTA, pH 13.1) for 30 min at 4 °C. Slides were placed side by side in electrophoresis unit (comet 20 system, Scie-Plas, Cambridge, England) attached with refrigerated water circulator (Julabo F12, Germany). Slides were immediately covered with freshly prepared alkaline electrophoresis buffer (300 mM NaOH, 1 mM Na_2_EDTA, pH 13.1) and electrophoresed (0.8 V/cm, 300 mA) using electrophoresis power supply (Consort EV261, Belgium) for 30 min at 4 °C. After completion of electrophoresis, slides were immersed in neutralizing buffer (0.4 M Tris, pH 8) twice for 5 min each. Finally, slides were dehydrated in 100 % methanol for 20 min and air dried following in an oven at 50 °C for 30 min. Slides were stained with 2.5 μg/mL propidium iodide and at least 500 comet cells were analysed using automated MetaCyte Comet Scan system (Metafer4, Zeiss, Germany).

### Protein extraction and western blot analysis in testes

The testes were dissected out at 2h, 4h, and 8h post-irradiation and homogenized in pre-chilled RIPA buffer (50 mMTris-Hcl, 150 mMNaCl, 0.5 % Sodium deoxicholate, 0.1 % SDS, 1 % Tween-100, 5 mM EDTA, 1 mM EGTA, 1 mM PMSF) containing protease inhibitor cocktail. Testes homogenates were centrifuged at 10,000 RPM for 15 min at 4 °C. Protein concentration was measured using Bradford method [41] with BSA standard curve. Equal quantity of protein were resolved by SDS-PAGE (12 % or 8 %) and transferred to PVDF membrane (Merck, Germany). Membranes were blocked with 5 % skimmed milk in TBST buffer (0.2 M Tris-base, 1.5 M NaCl, 0.1 % Tween-20) and incubated overnight with appropriate concentration of primary antibodies (ATM, p53, p21, Bax, Bcl-x, PCNA, active caspases-3, caspases-9 and β-actin) at 4 °C. Blots were washed and incubated with secondary antibody conjugated horseradish peroxidase for 1 h at room temperature. Secondary antibody bound membranes were washed with TBST buffer twice. Proteins bands were visualized using ECL chemiluminescence reagents and exposed to x-ray film. The intensities of each protein bands were analysed using Gel Doc XR (Bio-Rad, USA).

### Statistical analysis

The mean values and standard errors of the data were analyzed and reported. Pairwise comparisons were made between groups using Student’s t- test and ANOVA (Analysis of Variance). Statistically significant differences were considered among groups if the *P*-value was less than 0.05.

## Results

### Effect of melatonin on radiation-induced histopathological changes

**Fig. 1:**
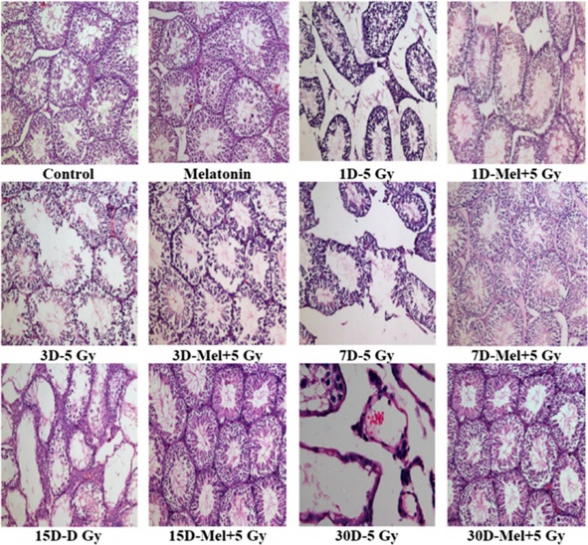
Effect of melatonin pre-treatment on the histological architecture of testes in mice exposed to whole-body ^60^Co γ-irradiation. Animals were sacrificed through cervical dislocation and testes were collected on 1^st^, 3^rd^, 7^th^, 15^th^ and 30^th^ days post-irradiation. After fixation and processing, cross sections of testes (5 μm) were stained with H & E and histological architecture of testes was analyzed. Representative photographs (1^st^ to 30^th^ Days) for testes histology are shown (original magnification 100X)

Whole-body γ-irradiation induces both qualitative and quantitative changes in the testes. Normal cellular association of spermatogenic cells (Spermatogonia, Sertoli Cell, Spermatocyte and Spermatid) in stepwise stage of development were observed in control and melatonin alone treated mice (Fig. 1). Radiation-induces (5 Gy) severe testicular atrophy with disorganization in the developmental stage and depleted spermatogenic cells, especially spermatogonia, spermatocytes and spermatid in seminiferous tubules on day three, but these changes in the architecture of testes were prominent on day seven post-irradiation (Fig. 1). Further, Leydig cells were found to be less between the tubules of irradiated mice (Fig. 1). Melatonin pre-treatment demonstrated relatively normal testicular architecture with regular cellular association and slight loss of spermatogenic cells in tubules on day three in comparison to irradiated mice (Fig. 1).

**Fig. 2:**
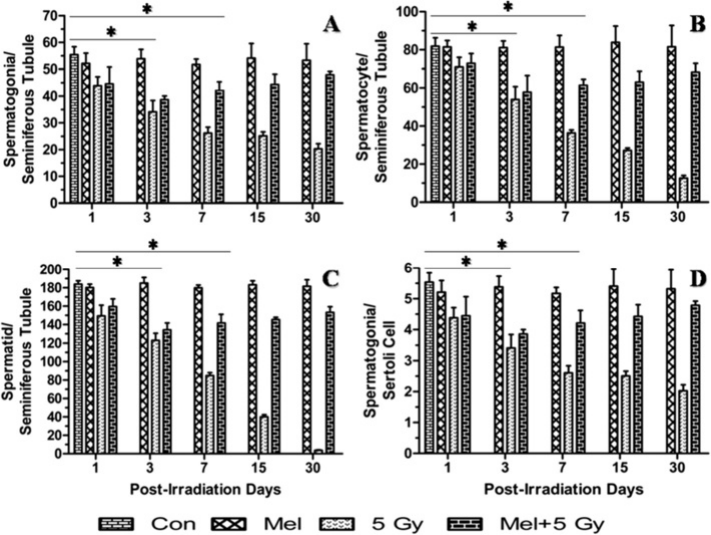
Effect of melatonin pre-treatment on spermatogenic cell in mice exposed to whole-body ^60^Co γ-irradiation. Animals were sacrificed through cervical dislocation and testes were collected on 1^st^, 3^rd^, 7^th^, 15^th^ and 30^th^ days post-irradiation. After fixation and processing, cross sections of testes (5 μm) were stained with H & E and spermatogenic cells were analyzed and represented. *p < 0.001

Radiation cause morphological changes in the testes histological architecture mainly due to the killing of spermatogonial cells [42], therefore, counting of spermatogonia is considered as gold standard to measure radiation-induced effects in testes. Spermatogenic cells were counted in seminiferous tubules to assess the γ-ray-induced testicular injury in mouse. A statistically significant (*p* < 0.001) reduction in spermatogenic cells (excluding sertoli cell) was observed on 3^rd^ day post-irradiation in irradiated mice (Fig. 2). Melatonin pre-treatment increased (*p* < 0.001) spermatogenic cells on 7^th^ day in comparison to radiation alone treated mice (Fig. 2). Sertoli cells appeared to be more radio resistant, therefore, did not show changes (*p* > 0.05) till 30^th^ day of observation (Additional file 1: Figure S1). Thus, the sertoli cells were considered as reference standard cells, only if they had nucleolus in the plane of section. The total number of spermatogonia was divided by total number of sertoli cells and expressed as ratio of spermatogonia/sertoli cells. The results showed that more number of spermatogonia per sertoli cell was present in melatonin pre-treated mice (*p* < 0.001) on 7^th^ day following irradiation (Fig. 2d). On the other hand, irradiation resulted a significant decrease in the number of spermatogonia per sertoli cell in irradiated mice (*p* < 0.001) as observed on 3^rd^ day (Fig. 2d). The present results suggest that a single prophylactic dose of melatonin recover spermatogenic cells in irradiated testes of mice as a function of post-irradiation days. Melatonin treatment alone did not show any change in the histological artchitechture and spermatogenic population of testes till 30^th^ day of observation (Figs. 1 and 2).

### Melatonin protects radiation-induced sperm abnormality, motility and viability

**Fig. 3:**
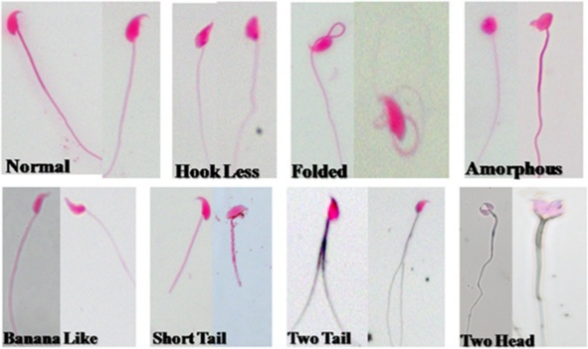
Morphologically classified sperm abnormalities in the cauda-epididymis of mice exposed to whole-body ^60^Co γ-irradiation. Animals were sacrificed through cervical dislocation and sperm was collected from cauda-epididymis. Single cell suspension of sperm was prepared in TNE buffer as well as prepared slide and stained with Eosin-Y. Seven different types of morphologically classified sperm abnormalities were observed and represented (original magnification 400X)

**Fig. 4:**
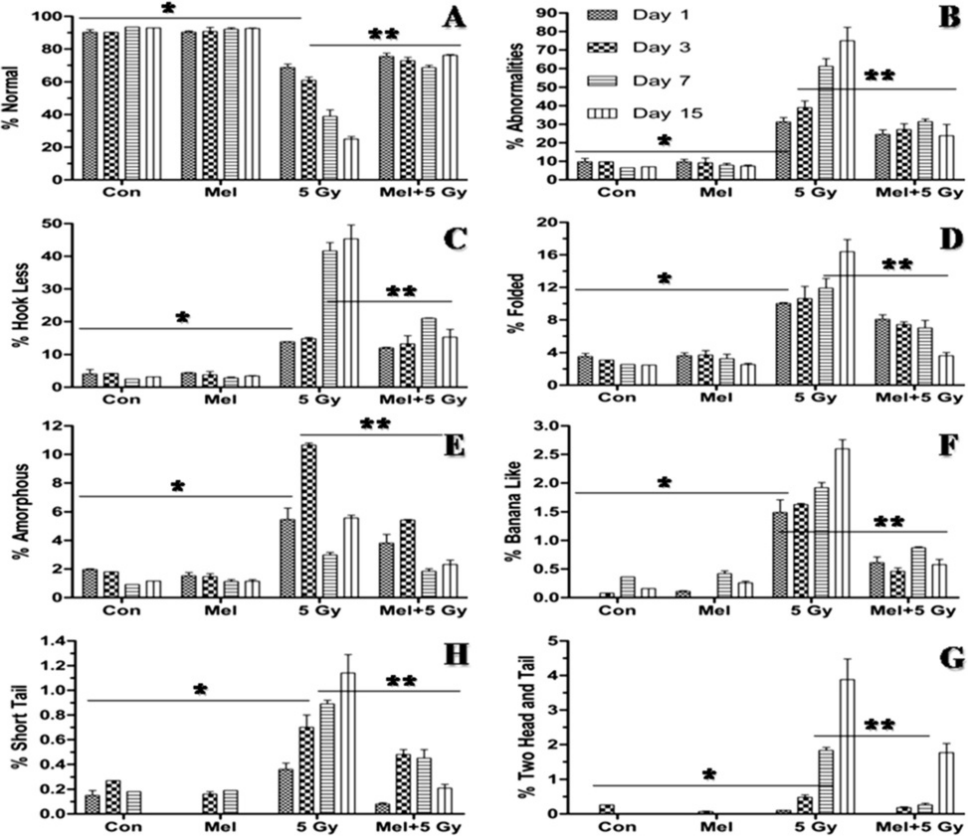
Effect of melatonin pre-treatment on sperm morphological abnormalities in mice exposed to whole-body ^60^Co γ-irradiation. Animals were sacrificed through cervical dislocation and sperm was collected from cauda-epididymis on 1^st^, 3^rd^, 7^th^ and 15^th^ days post-irradiation. Single cell suspension of sperm was prepared in TNE buffer as well as prepared slide and stained with Eosin-Y. *p < 0.001, **p < 0.001

The sperm morphology is important for direct assessment of sperm quality. Therefore, we have performed morphological evaluation of spermatozoa and analyzed seven types of sperm abnormalities in irradiated mice (Fig. 3). In the present study, a significant (*p* < 0.001) post irradiation day-dependent increase in total sperm abnormalities (expressed in percentage) viz., hook less, banana-like, amorphous, folded, short tail, two tail and two headed was observed on day one post-irradiation (Fig. 4b-h). Melatonin pre-treatment, however, significantly reduced (*p* < 0.001) % total sperm abnormalities (banana-like, amorphous, folded, short tail, two tail and two head) in comprison to irradiated mice on day three (Fig. 4b-h). Melatonin treatment alone did not appear to cause any sperm abnormalities (Fig. 4b-h). Percentage of two headed and two tails was found to very low, therefore, both were represented in a single parameter.

**Fig. 5:**
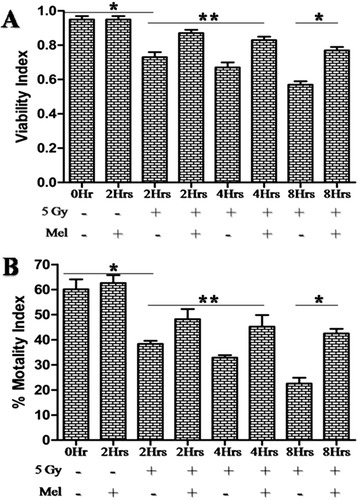
Effect of melatonin pre-treatment on sperm viability and motility in mice exposed to whole-body ^60^Co γ-irradiation. Animals were sacrificed through cervical dislocation and sperm was collected from cauda-epididymis after 2h, 4h and 8h post-irradiation. Sperm cells were stained with Eosin-Y and viable sperm cells were analyzed using haemocytometer. Immediately, after sacrificed, sperm cells motility was analyzed using haemocytometer. Results are represented as viability index and percentage motility index for viable and motile sperm cells, respectively. *p < 0.001, **p < 0.05

The motility and viability of sperms are important factors for normal functioning of sperm. Whole-body irradiation significantly decreased the motility and viability of sperm after 2h (*p* < 0.01), 4h (*p* < 0.01) and 8h (*p* < 0.001) post irradiation in comparison to control (Fig. 5a-b). Melatonin pre-treatment has significantly increased both the motility and viability of sperms at 2h (*p* < 0.05), 4h (*p* < 0.05) and 8h (*p* < 0.001) post irradiation. No significant changes were found in sperm motility and viability when melatonin alone treated mice were compared with control mice (Fig. 5a-b).

### Effect of melatonin on radiation-induced total antioxidant capacity

**Fig. 6:**
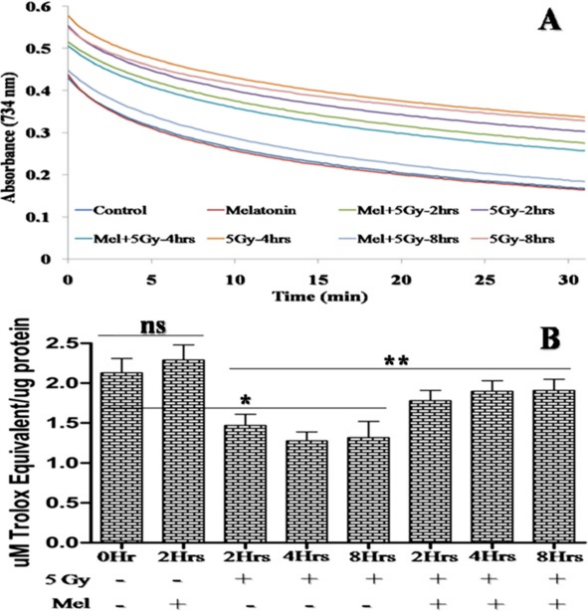
Effect of melatonin pre-treatment on TAC of testes in mice exposed to whole-body ^60^Co γ-irradiation. Animals were sacrificed through cervical dislocation and testes were collected after 2h, 4h and 8h post-irradiation. TAC of testes was measured in 10 % (w/v) homogenate through ABTS^•**+**^ decolorizing assay using spectrophotometer. The percentage inhibition of testes was equated with Trolox standard curve. TAC of testes was expressed as μM Trolox equivalent (TE) /μg protein. *p < 0.01, **p < 0.05, ns = non-significant (*p* > 0.05)

Testis has endogenous antioxidant defense system for maintaining the dual function viz., germ cells spermatogenic and Leyding cells steroidogenic functions [43]. TAC is crucial for countering radiation-induced oxidative damages in testes. Whole-body radiation exposure of 5 Gy induced marked decrease in TAC (*p* < 0.01) in comparison to control after 2h and remained 8h post-irradiation, indicating that imbalance between pro-oxidants and antioxidant leads to the overproduction of reactive oxygen species. However, pre-treatment of melatonin in irradiated mice significantly increased (*p* < 0.05) TAC at 2 to 8 h post-irradiation in comparison to radiation alone treated mice. The calculated value of TAC for testes in control mice was found to be 2.13 ± 0.18 μM TE/ μg protein (Fig. 6).

### Melatonin reduces radiation-induced lipid peroxidation

**Fig. 7:**
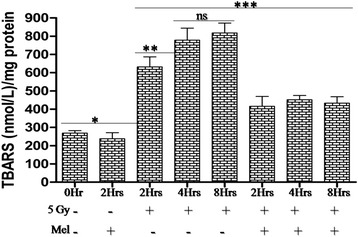
Effect of melatonin pre-treatment on lipid peroxidation of testes in mice exposed to whole-body ^60^Co γ-irradiation. Lipid peroxidation was analyzed after 2h, 4h and 8h post-irradiation in 10 % (w/v) homogenate prepared in pre-chilled PBS. Lipid peroxidation products in testes were measured using spectrophotometer and represented as TBARS (nmol/L)/ mg protein. *p < 0.001, **p < 0.01, ***p < 0.001, ns = non-significant (*p* > 0.05)

Lipid peroxidation is one of the critical events of ionizing radiation-induced oxidative damages. The level of TBARS significantly (*p* < 0.001) increased after 5 Gy whole-body radiation exposure among radiation alone treated mice. TBARS was further evaluated at 2h, 4h and 8h after 5 Gy radiation exposure, and demonstrated significant differences (*p* < 0.01) between 2 to 4 h and non-significant differences (*p* > 0.05) between 4-8h, suggesting that the TBARS increased significantly during the course of time till it reaches its maximum level at 4h. Melatonin pre-treatment significantly (*p* < 0.001) reduced TBARS level between 2 to 8 h post-irradiation in irradiated mice. This suggests that pre-treatment of melatonin decreased TBARS induced by ionizing radiation in normal mice testes (Fig. 7).

### Melatonin reduces radiation-induced DNA strands breaks

**Fig. 8:**
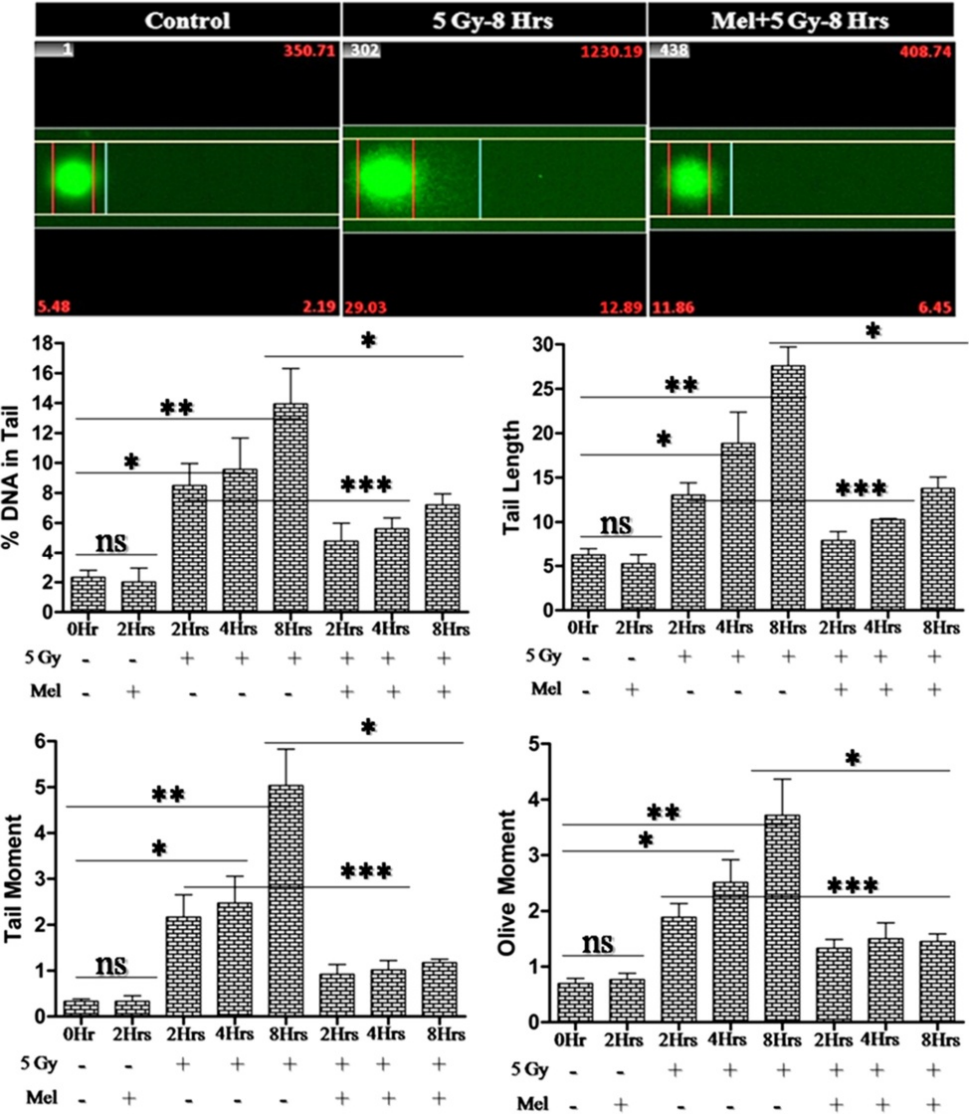
Effect of melatonin pre-treatment on DNA strands breaks of testes in mice exposed to whole-body ^60^Co γ-irradiation. An alkaline comet assay was performed to analyzed DNA strands breaks after 2h, 4h and 8h post-irradiation. DNA strands breaks are represented as tail length, tail moment, olive moment and % DNA in tail. *p < 0.01, **p < 0.001, ***p < 0.05, ns = non-significant (*p* > 0.05)

Maintenance of integrity of DNA in the germ cells is of utmost importance for reproduction, and therefore protection from free radical mediated DNA damages induced by γ-radiation is necessary. In the present study, we have assessed γ-irradiation induced DNA damages in germ cells by alkaline comet assay (Fig. 8). Whole-body γ-irradiation of 5 Gy increased DNA strands breaks parameters (tail length, tail moment, olive moment and % DNA in tail) significantly at 2h (*p* < 0.01), 4h (*p* < 0.01), and 8h (*p* < 0.001) post-irradiation in comparison to the control (Fig. 8). Melatonin pre-treatment reduced radiation-induced oxidative DNA strands breaks significantly at 2h (*p* < 0.05), 4h (*p* < 0.05), and 8h (*p* < 0.01) in comparison to radiation alone treated mice. The results suggest that melatonin pre-treatment provided significant protection to DNA against oxidative damages induced by γ-irradiation in normal testicular cell (spermatogenic cells) (Fig. 8).

### Effects of melatonin on the modulation of radiation-induced expression of ATM-dependent p53 pro-versus-anti apoptotic proteins

**Fig. 9:**
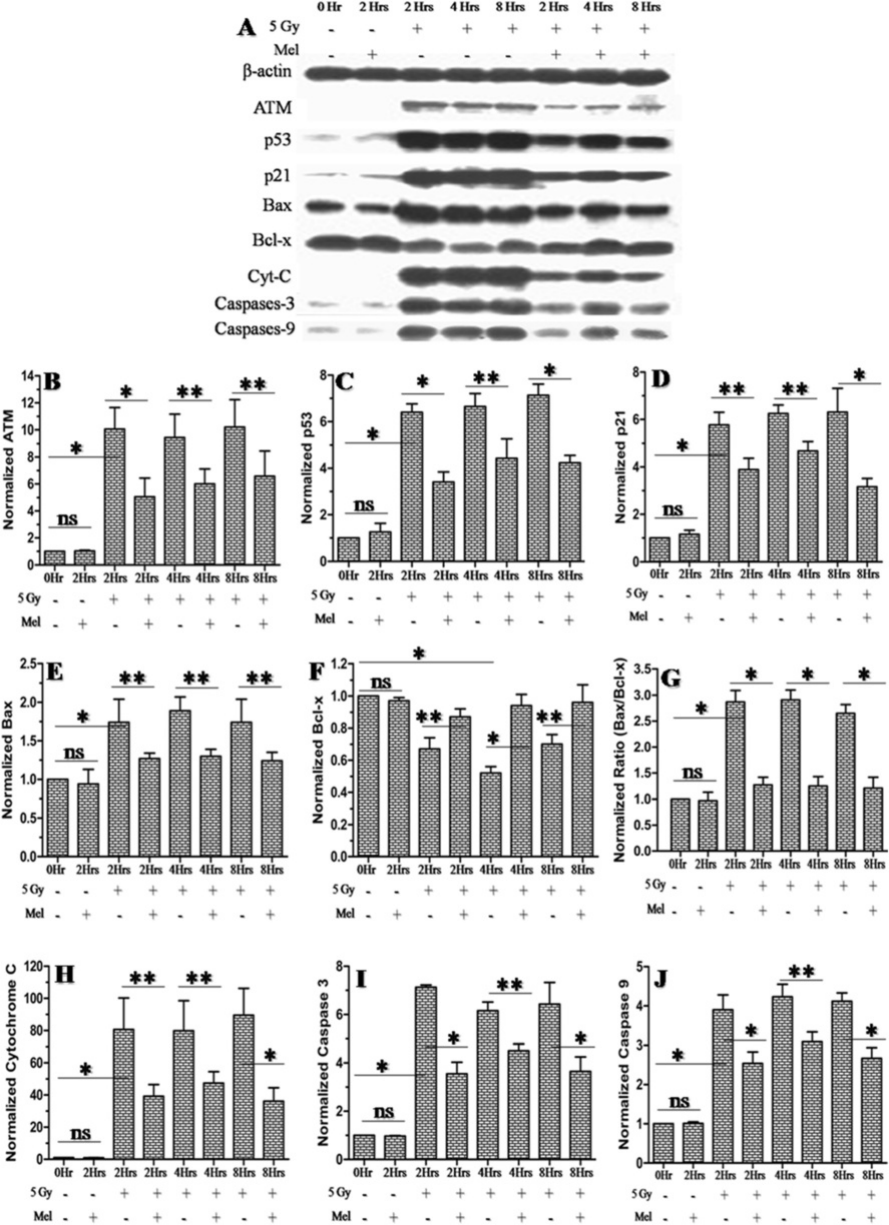
Effect of melatonin pre-treatment on anti and pro-apoptotic proteins expression of testes in mice exposed to whole-body ^60^Co γ-irradiation. Western blot was performed to measured both the anti (ATM, p53, p21, Bax, Bcl-x, cytochrome C, active caspases-3 and caspases-9 proteins) and pro (Bcl-xL)-apoptotic proteins expression after 2h, 4h and 8h post-irradiation. The β-actin protein was used as loading control. *p < 0.001, **p < 0.01, ns = non-significant (*p* > 0.05)

It is well established that ATM (ataxia telangiectasia mutated) directly activate p53 in response to DNA damage induced by ionizing radiation. p53 plays an important role in activating several pro-apoptotic signaling pathways, including p21, Bax, cytochrome C and active caspases-3 [44]. To assess the possible role of melatonin in regulating ATM dependent p53 apoptotic signalling proteins expression in testes of irradiated mice, we examined the expression pattern of ATM, p53, p21, Bax, Bcl-x, cytochrome C, active caspases-3 and caspases-9 proteins at 2h, 4h and 8h after 5 Gy whole-body γ-irradiation through western blotting. The cellular level of ATM protein was found to be up-regulated at 2h, 4h and 8h post- irradiation (5 Gy) and showed about 10-fold increase in comparison to the control (Fig. 9a-b). Melatonin pre-treatment inhibited (about 5-fold) the expression of radiation-induced ATM protein at 2h, 4h and 8h post-irradiation in comparison to radiation alone treated mice (Fig. 9a-b). Furthermore, the ATM dependent p53 apoptosis signalling pathway involving p53, p21, Bax, cytochrome C, active caspases-3 and caspases-9 were also up-regulated by irradiation (5 Gy) at 2h, 4h and 8h post-irradiation. The increase in protein expression was about 7-fold, except Bax and cytochrome C which showed about 2-fold and 80-fold, respectively (Fig. 9c, d, e, h, i and j). Pre-treatment with melatonin was found to inhibit the expression of these proteins about two to four-folds except for cytochrome C that decreased by about 40-folds (Fig. 9c, d, e, h, i and j). The results indicate that part of the radioprotective effect of melatonin may be due to the inhibition of expression of ATM-dependent p53-related apoptotic signalling proteins. We have also observed significant inhibition of anti-apoptotic Bcl-x protein at 2h, 4h, and 8h post-irradiation (5 Gy) in comparison to control (Fig. 9f). This decreased expression of Bcl-x protein resulted in a relatively increased Bax/Bcl-x ratio at all time points. This ratio (Bax/Bcl-x) was three to four-folds higher among irradiated mice in comparison to normal control (Fig. 9g). Melatonin pre-treatment decreased this up-regulated ratio of Bax/Bcl-x by two to three-folds in irradiated mice (Fig. 9g). Interestingly, melatonin alone treatment did not bring about any changes in the expression pattern of upstream regulators of apoptotic proteins (Fig. 9).

### Effect of melatonin on radiation-induced spermatogenic cell proliferation protein PCNA

**Fig. 10:**
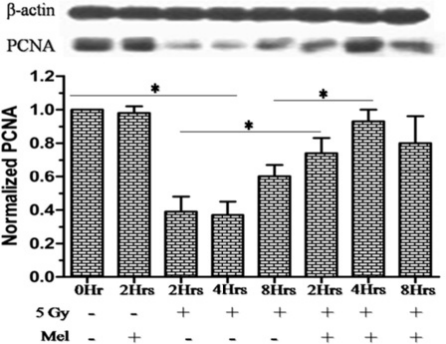
Effect of melatonin pre-treatment on PCNA protein expression of testes in mice exposed to whole-body ^60^Co γ-irradiation. Western blot was performed to measured PCNA protein expression after 2h, 4h and 8h post-irradiation. The β-actin protein was used as loading control. *p < 0.001

The progression of spermatogenic cell is of paramount importance for spermatogenic process, therefore, we have further evaluated the expression pattern of a nuclear protein and a co-factor for DNA polymerase δ, PCNA protein, which is reported to be involved in the RAD6-dependent DNA repair pathway in response to oxidative DNA damage. A significant decrease in PCNA protein was observed at 2h (*p* < 0.001), 4h (*p* < 0.001) and 8h (*p* < 0.01) in comparison to control, suggesting that the expression pattern of PCNA decreases during the course of time till it reaches the maximum level at 4h post-irradiation afterward increases (Fig. 10). Melatonin pre-treated mice displayed highly significant (*P* < 0.001) PCNA expression pattern in testes (Fig. 10). This indicated that melatonin pre-treatment increased PCNA protein expression, which was important for both the DNA repair and spermatogenic cell proliferation.

## Discussion

Protection of reproductive system against radiation-induced oxidative damage is of utmost importance during planned whole-body or partial-body radiation exposure scenarios. Therefore, development of prophylactic agent for medical management of radiation is necessary. In past few years, several compounds have been investigated for radioprotectors using both *in-vitro* and *in-vivo* model systems [45–47]. Toxicity and efficacy for human applications are still major concerns. Therefore, search for less or nontoxic and more efficacious radioprotector are continued for human use [48, 49].

Testis has endogenous antioxidant defense system comprising of highly structured arrangement of antioxidant enzymes, free radical scavengers, and low oxygen tension for maintenance of spermatogenic [43] and Leyding cells steroidogenic functions [43]. However, wide arrays of endogenous and exogenous factors including irradiation are known to disturb these defense systems and increase male infertility [50, 51]. Thus, radiation-induced male infertility in the event of planned radiation exposure countered by use of safe radioprotector.

In an earlier study, carbon-ion radiation exposure (high-LET) caused cellular perturbation includes, marked changes in histopathology, increased lipid peroxidation, DNA strand breaks, chromosome aberrations, apoptosis and imbalance antioxidant status, as well as inactivation of PARP-1(DNA repair enzymes) in mouse testis [52]. Melatonin pre and post treatment decreased carbon-ion radiation-induced histopathological changes, DNA strands breaks, lipid peroxidation and apoptosis in association with increase of GSH and TAC [53]. Further, carbon ion beams also induced harmful effects on pre and postnatal testicular developmental stages. It has been observed that, when the abdomen of pregnant rat was irradiated on gestation day 15, breeding activity of male offspring was affected [54]. In comparison to high-LET irradiation, low-LET mediated damage is thought to mostly through generating highly reactive free radicals. To the best of our knowledge, no study in literature has reported radioprotection by melatonin against low-LET mediated mouse testicular injury. Few histopathological studies [23–25] suggested that melatonin reduced low-LET mediated rat testicular injury. These preliminary studies [23–25] and reports of radioprotection by Vijayalaxmi et al., [12–22] have prompted us to undertake a detailed investigation of radioprotective potential of melatonin in amelioration of acute testicular injury induced by γ-irradiation in murine model. We have investigated effect of melatonin in amelioration of acute testicular injury induced by γ-irradiation in mice. Our results have shown that melatonin pre-treatment attenuated γ-ray-induced acute testicular injury in mouse testes indicated by restoring the spermatogenic cells population in seminiferous tubules (Figs. 1 and 2) in association with increase of sperms viability as well as motility (Fig. 5) and decrease of sperms abnormalities (Figs. 3 and 4). These ameliorating effects of melatonin were due to its ability to increase the level of TAC (Fig. 6) together with decrease of lipid peroxidation (Fig. 7) and DNA strands breaks (SSBs, DSBs and alkali-labile lesions) (Fig. 8) in irradiated mice testes.

Elucidation of the molecular mechanism of radioprotective drugs is necessary and required for new drug approval process. To the best of our knowledge, no study has reported molecular mechanism of melatonin in amelioration of γ-ray-induced mouse testicular injury. Therefore, the molecular mechanism underlying melatonin-testicular cytoprotection in irradiated mice was studied. Ionizing radiation-induces ATM-dependent p53 activation in response to DNA strands breaks. The gene p53 plays a vital role in the activation and mobilization of several pro/ anti-apoptosis markers include ATM, p53, p21, Bax, Bcl-x, cytochrome C, active caspases-3, caspases-9 and others [55–60]. Among these, Bax induces apoptosis by mobilization of Bax to mitochondria through p53. In the mitochondrial membrane, Bax oligomers accumulation cause release of cytochrome C, this led to caspases activation (cysteine-aspartic acid proteases). The release of cytochrome C can stop electron transfer leading to loss of mitochondrial membrane potential and ATP generation [59, 61]. The anti-apoptotic marker, Bcl-2 has been shown to counter the action of Bax and therefore prevent apoptosis. The expression of pro versus anti-apoptotic proteins may decide the sensitivity of cells towards apoptosis. The balanced Bax/Bcl-2 proteins ratio (pro versus anti-apoptotic proteins ratio), therefore, is of great significance for cell survival [62, 63]. Our western blot results indicate that melatonin pre-treatment inhibited radiation-induced expression of ATM-dependent p53 pro-apoptotic markers, ATM, p53, p21, Bax, cytochrome C, active caspases-3 and caspases-9 (Fig. 9). This decreased expression of pro-apoptotic proteins were associated with the increase of anti-apoptotic Bcl-x protein leading to balanced Bax/Bcl-x ratio in melatonin pre-treated mice (Fig. 9f-g).

Progression of cell cycle is important for proliferating spermatogenic cells. The proliferating cell nuclear antigen (PCNA) was originally identified as a nuclear antigen in proliferating cells. Subsequently, this protein described as a cofactor for DNA polymerase δ, which involved in the control of DNA replication and repair. PCNA protein levels rise only during the S-phase of the cell cycle, and form complex with p21 inhibitor. PCNA protein ubiquitinated and involved in the RAD6-dependent DNA repair pathway in response to oxidative DNA damage. In the present study, melatonin pre-treatment enhanced the expression of PCNA protein in response to irradiation-induced DNA damage (Fig. 10).

## Conclusions

Melatonin pre-treatment alleviated TAC and inhibited γ-ray-induced lipid peroxidation and DNA strands breaks in testes of γ-irradiated mice. Radiation-induced spermatogenic cells depletion in seminiferous tubules as well as sperm abnormalities, motility and viability in cauda-epididymis were markedly prevented by melatonin pre-treatment. Melatonin pre-treatment inhibited ATM-dependent p53 apoptotic signaling proteins- ATM, p53, p21, Bax, cytochrome C, active caspases-3 and caspases-9. The inhibition of apoptotic proteins was associated with the increase of anti-apoptotic-Bcl-x proteins. In addition, melatonin pre-treatment also protected RAD6 DNA repair-PCNA protein. These results clearly suggest prophylactic implication of melatonin in amelioration of low-LET mediated testicular injury in mouse. These results will be useful for understanding the radioprotective potential of melatonin in male reproductive system and, can be exploited for its use in cancer radiotherapy patients undergoing hemibody and abdominopelvic region radiation exposure.

## Additional file

Additional file 1: Figure S1. Effect of melatonin pre-treatment on sertoli cells in mice exposed to whole-body ^60^Co γ-irradiation. Animals were sacrificed through cervical dislocation and testes were collected on 1^st^, 3^rd^, 7^th^, 15^th^ and 30^th^ days post-irradiation. After fixation and processing, cross sections of testes (5 um) were stained with H & E and sertoli cells were analyzed and represented. ns = non-significant.
