# Correction: Radioprotective potential of melatonin against ^60^Co γ-ray-induced testicular injury in male C57BL/6 mice

**DOI:** 10.1186/s12929-022-00843-w

**Published:** 2022-11-02

**Authors:** Shahanshah Khan, Jawahar Singh Adhikari, Moshahid Alam Rizvi, Nabo Kumar Chaudhury

**Affiliations:** 1 Chemical Radioprotector and Radiation Dosimetry Research Group, Division of Radiation Biosciences, Institute of Nuclear Medicine and Allied Sciences, Defence Research & Development Organization, Brig. S. K. Mazumdar Road, New Delhi, 110054 India; 2 Genome Biology Laboratory, Department of Biosciences, Faculty of Natural Sciences, Jamia Millia Islamia, New Delhi, 110025 India

## Correction: Journal of Biomedical Science (2015) 22:61 10.1186/s12929-015-0156-9

In the original article [1], the image corresponding to the group (30D-Mel-5 Gy) was partially similar with another image (15D-Mel-5 Gy) in Fig. 1. This error has occurred due to inadvertent selection of image during arranging of all images of Fig. 1. These images are replaced by correct images from respective groups stored image files. the complete histological (H&E slides) evaluation as a routine practice was performed in a blinded manner (all slides were code) and therefore this correction has not caused any changes in quantitative and qualitative analyses.

**Fig. 1:**
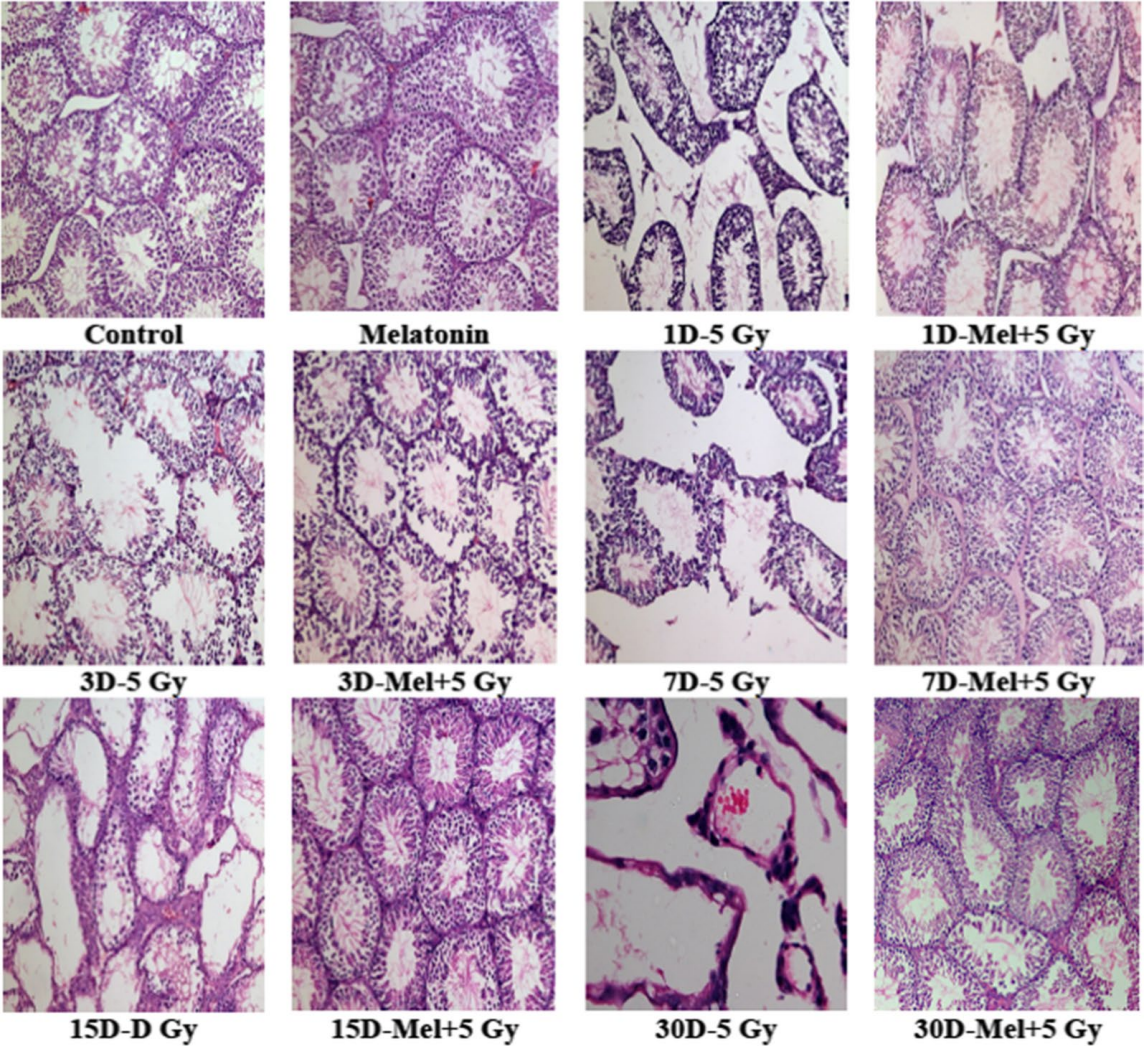
Effect of melatonin pre-treatment on the histological architecture of testes in mice exposed to whole-body ^60^Co γ-irradiation. Animals were sacrificed through cervical dislocation and testes were collected on 1st, 3rd, 7th, 15th and 30th days post-irradiation. After fixation and processing, cross sections of testes (5 μm) were stained with H&E and histological architecture of testes was analyzed. Representative photographs (1st to 30th days) for testes histology are shown (original magnification ×100)

The inadvertent error of misplacing the image in Fig. 1 is regretted.
