# Supplementary figures and images for: Radioprotective potential of melatonin against 60Co γ-ray-induced testicular injury in male C57BL/6 mice
Source: J Biomed Sci. 2015 Jul 24;22(1):61. doi: 10.1186/s12929-015-0156-9 (PMC4514449; doi:10.1186/s12929-015-0156-9)

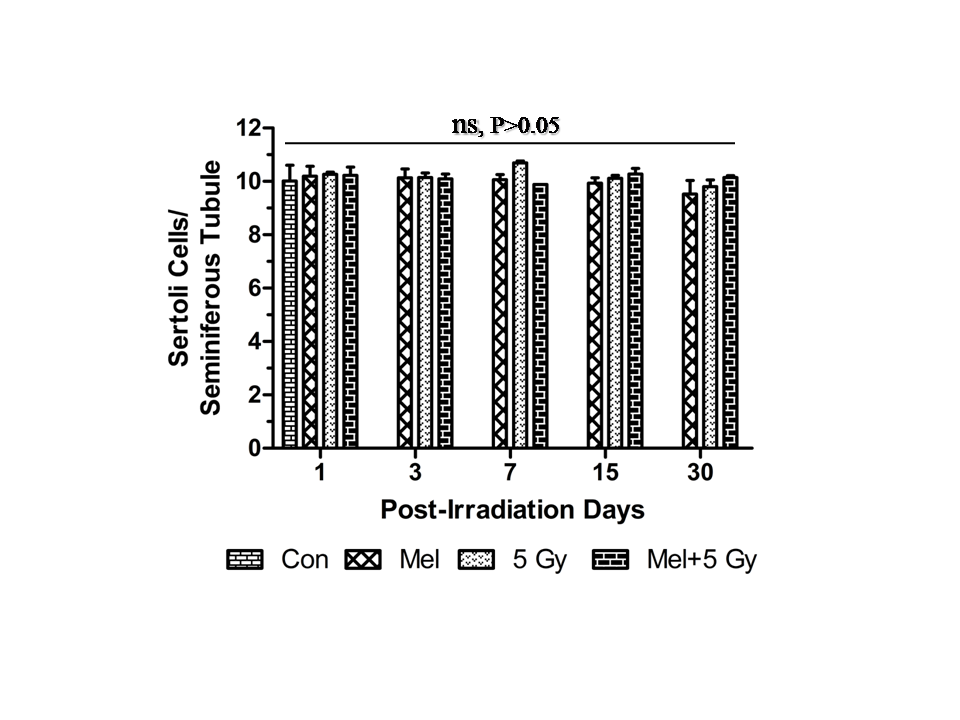

Supplement: Additional file 1: Figure S1. — Effect of melatonin pre-treatment on sertoli cells in mice exposed to whole-body 60Co γ-irradiation. Animals were sacrificed through cervical dislocation and testes were collected on 1st, 3rd, 7th, 15th and 30th days post-irradiation. After fixation and processing, cross sections of testes (5 um) were stained with H & E and sertoli cells were analyzed and represented. ns = non-significant. [file 12929_2015_156_MOESM1_ESM.tif]
